# Tie2-Dependent Neovascularization of the Ischemic Hindlimb Is Mediated by Angiopoietin-2

**DOI:** 10.1371/journal.pone.0043568

**Published:** 2012-09-25

**Authors:** Michael Lekas, Poli Lekas, Shirley H. J. Mei, Yupu Deng, Daniel J. Dumont, Duncan J. Stewart

**Affiliations:** 1 The Terrence Donnelly Research Laboratories, Division of Cardiology, St. Michael's Hospital and the Department of Medicine, University of Toronto, Ontario, Canada; 2 Heart and Stroke/Richard Lewar Centre of Excellence, Faculty of Medicine, University of Toronto, Canada; 3 Molecular and Cellular Biology Research, Sunnybrook Research Institute, Toronto, Ontario, Canada; 4 Ottawa Hospital Research Institute and the University of Ottawa, Ontario, Canada; University of Illinois at Chicago, United States of America

## Abstract

The angiopoietins (ANGPT) are ligands for the endothelial cell (EC) receptor tyrosine kinase, Tie2. Angpt-1 is a Tie2 agonist that promotes vascular maturation and stabilization, whereas Angpt-2 is a partial agonist/antagonist involved in the initiation of postnatal angiogenesis. Therefore, we hypothesized that overexpression of Angpt-2 would be more effective than Angpt-1 for enhancing the perfusion recovery in the ischemic hindlimb. Perfusion recovery was markedly impaired in Tie2-deficient animals at day 35 in a model of chronic hindlimb ischemia. Injections of *Angpt-2* or *VEGFA* plasmid at 7 days post femoral artery resection enhanced recovery and improved arteriogenesis as assessed by angiographic scores, whereas *Angpt-1* or null plasmid had no effect. In addition, Angpt-2 together with VEGF resulted in greater improvement in perfusion and collateral vessel formation than VEGF alone. Similarly, conditional overexpression of Angpt-2 in mice improved ischemic limb blood flow recovery, while Angpt-1 overexpression was ineffective. These data from Tie2 heterozygote deficient mice demonstrate, for the first time, the importance of the Tie2 pathway in spontaneous neovascularization in response to chronic hindlimb ischemia. Moreover, they show that overexpression of the partial agonist, Angpt-2, but not Angpt-1, enhanced ischemic hind limb perfusion recovery and collateralization, suggesting that a coordinated sequence antagonist and agonist activity is required for effective therapeutic revascularization.

## Introduction

The endothelial-selective receptor tyrosine kinase (RTK), Tie2, plays an essential role in blood vessel formation during embryonic development [1]. Targeted deletion of Tie2 [2] or its major agonist ligand, angiopoietin 1 (Angpt-1) [3], results in embryonic lethality in mice characterized by defects in blood vessel maturation, lack of recruitment of supporting pericytes and impaired basement membrane formation [3], and embryonic loss occurs at a slightly later stage than for mice deficient in vascular endothelial growth factor-A (VEGF) or its receptor, VEGFR2 [4]. Thus, VEGF and Angpt-1 appear to function in a temporally segregated yet complimentary manner in the blood vessel formation in the developing embryo [5], [6], [7]; however, the role of the angiopoietin system in postnattal angiogenesis is less clear.

Angiopoietin-2 (Angpt-2) is another major Tie2 ligand. While both Angpt-1 and Angpt-2 bind to Tie-2 with equal affinity [8], Angpt-2 has been characterized as a functional antagonist of Tie2 [8], blocking the effects of Angpt1 on Tie2 activity. The finding of increased Angpt-2 expression at the leading edge of tumour neovessels [9] has led to the concept that Angpt-2 is required to release endothelial cells (EC) from the tonic inhibitory effect of Angpt-1 and facilitate EC activation in response to VEGF [10]. Moreover, in the absence of VEGF, Angpt-2 has been shown to promote EC apoptosis [11] and has been implicated in mediating vascular regression in the involuting corpus luteum [12]. However, it has recently been recognized that Angpt-2 exhibits context-dependent agonist activity, inducing activation of Tie-2 in a time-dependent manner to levels similar to Angpt-1 at high concentrations [13] or during prolonged (i.e. 12 to 24 hours) exposure [14], which corresponds to the time course of capillary-like network formation in cultured ECs [14]. These findings point to a possible role for Angpt-2, not only as an inhibitor of Tie2 in the initiation of the angiogenic response, but also as an agonist in the later stages of blood vessel formation and maturation that are dependent on Tie-2 activation [7].

Previously, there have been conflicting reports on the role of the angiopoietins in postnatal angiogenesis and neovascularization. In the corneal implant model, Angpt-1 was shown to enhance neovessel density in combination with VEGF, but had no effect by itself, whereas Angpt-2 increased length but not the density of neovessels [15]. Similarly, synergistic effects of Angpt-1 and VEGF were seen in the ischemic hindlimb model [16], whereas, Angpt-2 was reported to impair angiogenesis in the same model [17]. However, others have reported that Angpt-2 is highly expressed in vascular regions undergoing active angiogenesis [18] and plays a requisite role in postnatal angiogenic vascular remodeling [19]. Moreover, it was recently shown that the selective inhibition of Angpt-2 activity impaired recovery of blood flow in the ischemic hind limb [20], consistent with an important role for the endogenous ligand in angiogenesis and collateral vessel formation in this model.

In the present study, we show for the first time that Tie2 deficiency resulted in exacerbation of limb loss and impaired spontaeous perfusion recovery in the context of hindlimb ischmeia. Moreover, overexpression of Angpt-2, but not Angpt-1, markedly enhanced collateral formation in the rat hindlimb ischemia model, which was further augmented by combination with VEGF. As well, induction of Angpt-2 in conditional transgenic mice also increased circulating levels of progenitor cells. These data strongly support the predominant role of Angpt-2 in postnatal angiogenesis and collateral vessel formation.

## Results

### Tie2 Deficiency Results in Increased Limb Necrosis and Impaired Perfusion Recovery

**Figure 1:**
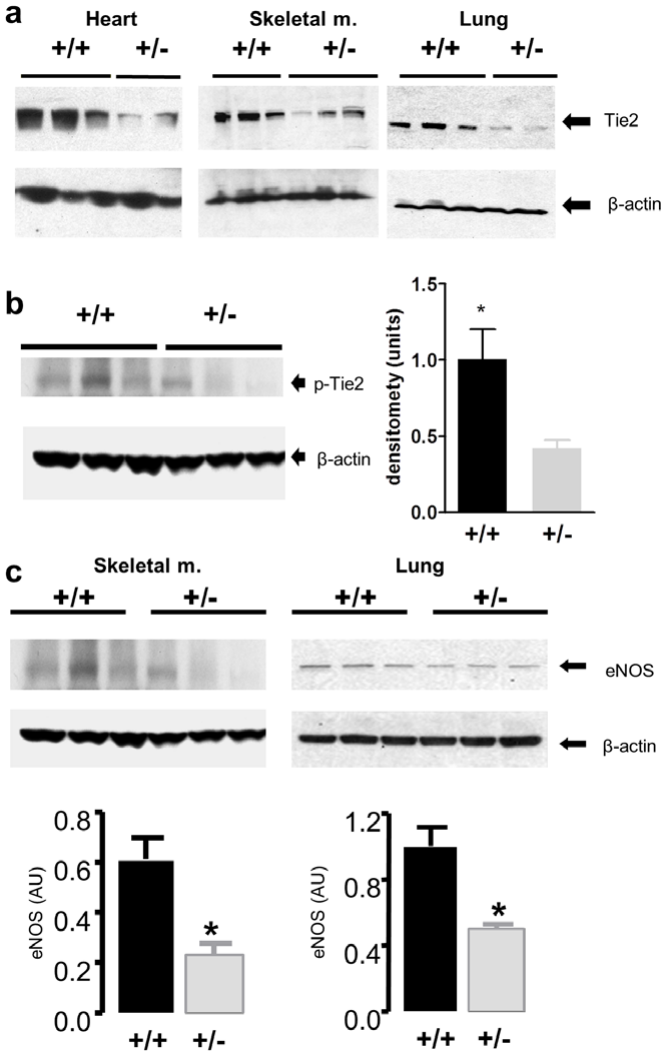
Tie2 heterozygote deficient mice (Tie2+/−) express less total Tie2 and phosphorylated Tie2 (p-Tie2) content compared to wild-type mice in the highly vascularized beds of skeletal muscle, heart and lung. (a) Western immunoblots showing Tie2 (140 kDa) and β-actin (47 kDa) expression in Tie2 heterozygote deficient (Tie2+/−) and wild-type (WT) mouse heart, skeletal muscle and lungs. (b) Western immunoblot of phophorylated Tie2 (140 kDa) and β-actin from non-ischemic skeletal muscle expressed as a ratio of p-Tie2 to β-actin. * denotes p<0.05 vs. Tie2+/−. (c) eNOS levels are decreased in Tie2+/− compared to wild-type. Western immunoblots showing eNOS and β-actin in mouse hindlimb adductor muscle (left) and lung (right) *in vivo*. eNOS and β-actin were expressed as 133 and 47 kDa bands, respectively. Densitometric scanning of blots was used to determine levels of eNOS protein expressed as relative to β-actin. * denotes p<0.05 vs. WT.

**Figure 2:**
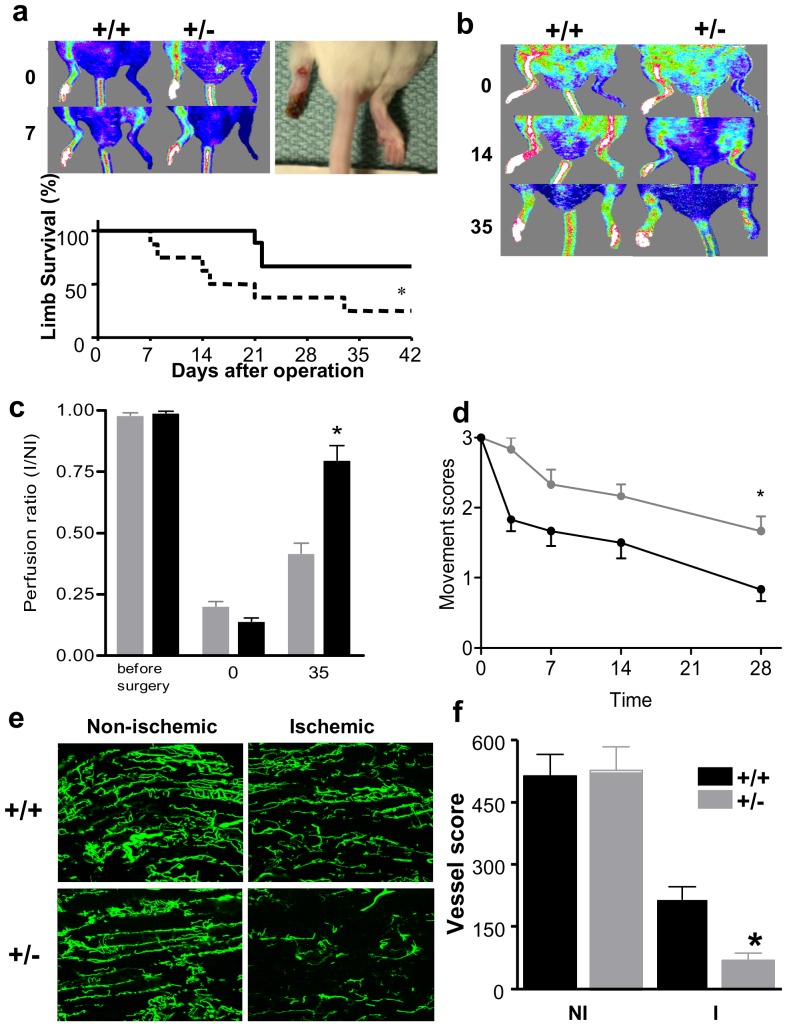
Limb survival dependence on Tie2 in the autoamputation model. (a) Representative laser doppler perfusion images showing post-surgical, unilateral reduction of hindlimb perfusion on day 0 (top) with signs of limb salvage in wild-type and early limb loss in Tie2+/− mice on day 7 (bottom). In color-coded images, normal perfusion is depicted in a range from high (red/white) to low (blue). Photographic image of distal forefoot loss defined as leg autoamputation. Graph shows limb survival curve after induction of ischemia (time 0) in wild-type (^___^, n = 9) and Tie2+/− (—, n = 8) mice as obtained by Kaplan-Mayer's method and analyzed using log-rank test. *denotes p<0.05. (b) Tie2+/− mice display reduced perfusion restoration compared to wild-type mice by laser doppler perfusion imaging in wild-type (n = 10) and Tie2+/− (n = 11) mice on day 0, 14 and 35 after femoral arteriectomy. (c) Quantitative evaluation of perfusion expressed as a ratio of ischemic to non-ischemic limb perfusion in wild-type (solid bars) and Tie2+/− mice (shaded bars); *denotes p<0.001. (d) Movement score assessing functional impairment in follow-up after hindlimb ischemia in wild-type (solid lines, n = 6) and Tie2+/− (shaded lines, n = 6) mice. *denotes p<0.05. (e) Tie2 deficiency decreases restoration of microvascular perfusion capacity in response to ischemia imaged with confocal microscopy on whole-mount tissue specimens using fluorescence microangiography. Nonischemic muscle in both Tie2+/− and wild-type contain numerous perfused blood vessels that dramatically drop in number after surgical induction of hindlimb ischemia. (f) Quantification of adductor muscle perfusion capacity on day 14 expressed as a vessel score that is a composite of vessel branching point number, length and end points number. Perfusion capacity of the ischemic limb is improved in wild-type (n = 5) versus Tie2+/− (n = 4) mice on day 14. *denotes p<0.01.

Tie2 protein and activity was decreased by 40–50% in Tie2^+/−^ versus Tie2^+/+^ mice (Figure 1a and b). Interestingly, eNOS protein expression was also reduced in Tie2-deficient animals (Figure 1c). Using the critical ischemia model, we tested the functional importance of Tie2 deficiency on limb survival. Wide surgical excision of the femoral artery produced immediate and profound reduction of hindlimb perfusion at day 0 and critical limb ischemia in both Tie2^+/+^ and Tie2^+/−^ animals (Figure 2a), with early (day 7) signs of tissue necrosis and distal forefoot loss were observed in Tie 2 deficient mice, associated with signficantly reduced perfusion by LDPI. Tie2+/− animals also exhibited a greater incidence of limb loss compared to Tie2+/+ animals at 42 days (75% versus 33%, p<0.05).

Femoral ligation with limited excision resulted in a stable model of chronic hindlimb ischemia without evidence of limb necrosis and tissue loss in either Tie2-deficient or WT mice. Hindlimb perfusion, assessed by serial LDPI measurements over 5 weeks, was reduced immediately after surgery in both Tie2+/+ and Tie2+/− mice (0.14±0.03 and 0.20±0.02, respectively) (Figure 2b). Recovery of perfusion in the ischemic (I) vs. the nonoperated (NI) limb was reduced in Tie2 deficient compared to WT mice at day 35 (I/NI 0.42±0.04 vs.0.79±0.06, respectively; p<0.001) (Figure 2c). Similarly, the active use of the ischemic limb was impaired after surgery, as reflected by an increase in the Movement Score [21], [22] (Figure 1d), which improved more rapidly and completely in the WT compared to Tie2 deficient animals (day 28: 0.8±0.20 vs. 1.67±0.21, respectively, p<0.05).

**Figure 3:**
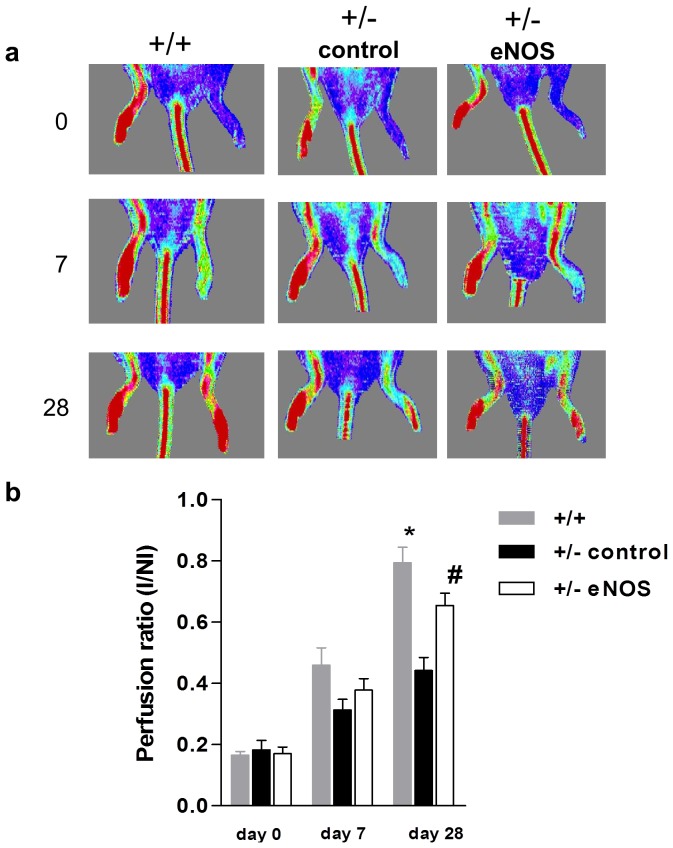
Intramuscular injection of plasmid-encoded eNOS augments perfusion restoration in the ischemic hindlimb of Tie2+/− mice. (a) Representative laser doppler perfusion imaging scans of WT (+/+, n = 5, left panel) and Tie2 deficient mice (+/−). Tie 2+/− mice received either intramuscular injections of plasmid encoding for human eNOS (n = 4, right panel) or null plasmid (n = 5, controls, middle panel). Serial imaging performed on days 0, 7 and 28 after surgically-induced left hindlimb ischemia. (b) Serial quantitative evaluation of hindlimb perfusion expressed as a ratio of ischemic to non-ischemic limb (I/NI) perfusion in wild-type (gray bars) and eNOS treated Tie2+/− (white bars) and control mice (black bars); *denotes p<0.001 WT vs. control; and # denotes p<0.05 eNOS vs. control. Blood flow is displayed by color-coded pixels where blue indicates low perfusion and red denotes high perfusion.

Interestingly, intramuscular injection (day 7) of plasmid-encoded eNOS into the ischemic hindlimb of Tie2 deficient mice enhanced perfusion recovery in comparison to control (Tie2+/−) mice treated with null plasmid (0.66±0.04 vs. 0.44±0.03, respectively, p<0.05) (Figure 3) to levels similar to WT (0.79±0.05).

### Tie2 Deficient Mice Display Diminished Microvascular Perfusion

Fluorescent microangiography revealed similar high levels of microvascular perfusion in the non-ischemic limb in WT and Tie2-deficient animals (Figure 2e). In contrast, there was a profound reduction in microvascular perfusion 14 days after surgery in ischemic limbs, which was more pronounced in the Tie2-deficient animals. Vessel score was determined using morphometric software as a composite of vessel length, nodal branching points and vessel segment end numbers (Figure 2f). Fourteen days after surgery, the hindlimb vessel score was profoundly decreased in both Tie2+/+ and Tie2+/− mice; however, this score was greater in Tie2+/+ than in Tie2+/− mice after 14 days (217.2±29.4 vs 69±18.5, respectively; p<0.01) indicative of a less recovery of deep muscle perfusion in the Tie2 deficient model.

### Spatio-temporal expression profile of Angiopoietins in the ischemic hindlimb

**Figure 4:**
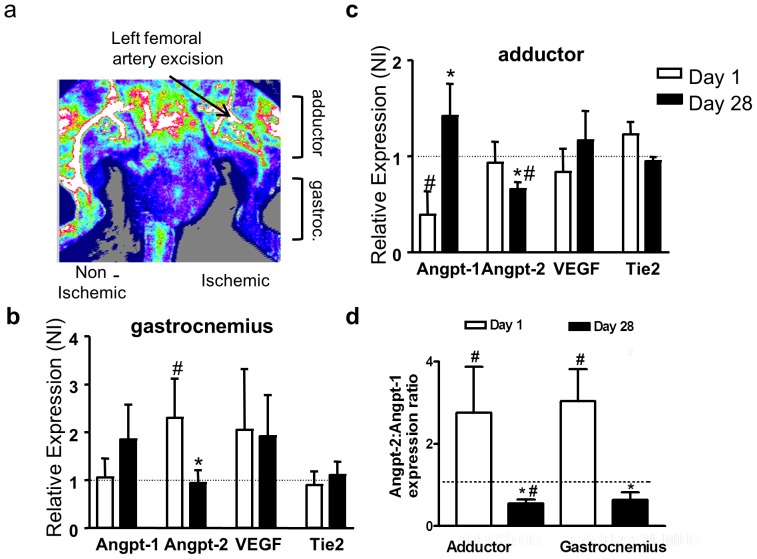
Changes in Angpt-1, Angpt-2, Tie-2 and VEGF mRNA expression in the rat hindlimb in the early (day 1) and late (day 28) stages of neovascularization in response to surgical induction of hindlimb ischemia by extensive femoral artery excision. (a) Laser doppler perfusion image of exposed upper leg in the anesthetized rat 28 days post-operative induction of hindlimb ischemia. Arteriogenesis or collateral growth occurs in the adductor muscle region of the ischemic leg bypassing the site of femoral artery excision to provide perfusion to the distal leg gastrocnemius muscle. Note strong signal in thigh of ischemic leg and poor signal in thigh of non-ischemic leg. Perfusion flows range from high (white) to low (blue) color. Summary data of semi-quantitative RT-PCR results from samples of gastrocnemius muscle (b), a site of angiogenesis; the proximal adductor muscle (c), site of collateral artery growth, obtained from the operated, ischemic limb and the contralateral non-operated, non-ischemic limb at 1 (n = 5) and 28 (n = 4) days after ischemia induction. mRNA expression of all genes was normalized to HPRT. Relative mRNA expression was derived as a ratio of ischemic to contralateral, non-ischemic limb (I/NI) expression. # denotes p<0.05 in comparison to unity (dashed line). * indicates p<0.05 in mRNA expression between day 1 and day 28. (d) The ratio of Angpt-2∶Angpt-1 expression was calculated for both gastrocnemius and adductor muscle samples at day 1(n = 5) and day 28 (n = 4–5). # denotes p<0.05 in comparison to unity (dashed line). * indicates p<0.05 in mRNA expression between day 1 and day 28. In all panels the dashed line at unity denotes equal mRNA expression in the ischemic and non-ischemic contralateral limb.

Endogenous mRNA expression of Angpt-2 was found to be increased in the ischemic gastrocnemius muscle at day 1 (Figure 4a and b), returning to baseline expression levels by day 28 as the endogenous blood flow recovery reached a plateau. In contrast, there was a reciprocal change in regulation of Angpt-1 expression, which was unchanged or even reduced initially and increased at later time periods, particularly in more proximal regions of the limb (i.e. adductor muscle) (Figure 4c). Despite these differences in expression profile, there was a parallel change in the ratio of Angpt-2/Angpt-1, which was increased by more than 2-fold at day 1, and reduced by about 50% at day 28, in both the ischemic gastrocnemius and the more proximal adductor muscles (Figure 4d). However, in the adductor muscle the early increase in ration was due mainly to a reduced expression of Angpt1, whereas in the gastrocnemius it was driven mainly by an increase in Angpt-2.

### Angpt-2 gene transfer enhances perfusion in response to ischemia

**Figure 5:**
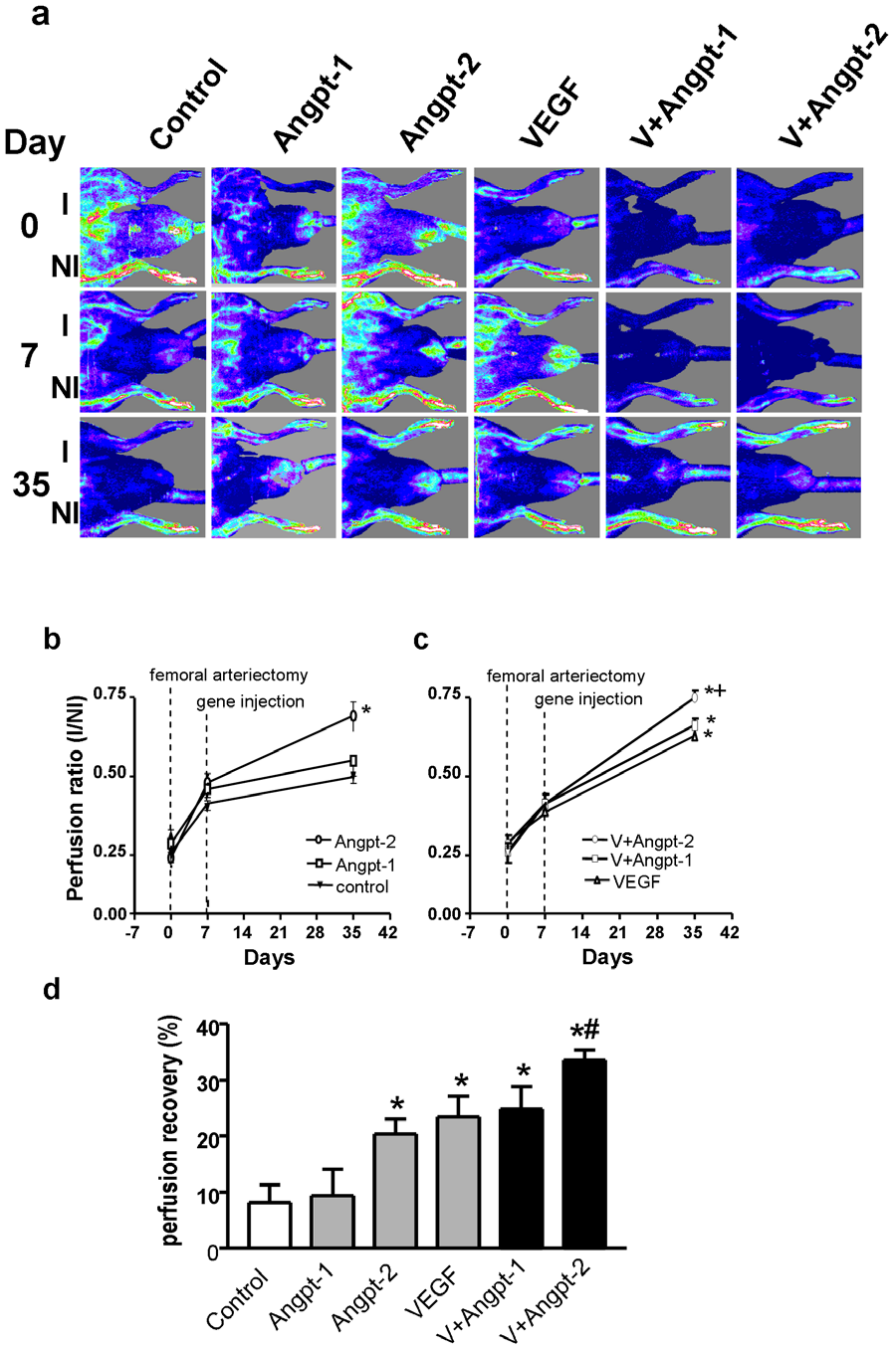
Laser doppler perfusion imaging serial assessment of hindlimb perfusion. (a) Representative laser doppler perfusion imaging scans of rats treated with intramuscular injections of plasmids encoding for human Angpt-1 alone (-□-, n = 7), Angpt-2 alone (-○-, n = 8), VEGF alone (-Δ-, n = 9) or combination of Angpt-1+VEGF (-□-, n = 8), Angpt-2+VEGF (-○-, n = 7). Controls received null plasmid (-Δ-, n = 9). Animals were imaged on days 0, 7 and 35 after surgically-induced left hindlimb ischemia. Blood flow is displayed by color-coded pixels where blue indicates low perfusion and red-white denotes high perfusion. Assessment of perfusion was performed on the lower leg and foot. (b) A serial quantitative evaluation of hindlimb blood perfusion expressed as a ratio of ischemic leg perfusion to that in the non-ischemic contralateral limb (I/NI) is shown for all single gene transfer groups. Combination gene transfer groups are shown in (c). (d) The increment in perfusion recovery (%) from day 7 (time of gene injection) to day 35. ^*^ denotes p<0.05 versus controls and Angpt-1 treated. # denotes p<0.05 versus VEGF and Angpt-2 treated animals.

Figure 5a shows representative images of hindlimb perfusion before (day 0), just prior to intramuscular delivery of null (control) plasmid or Angpt-1, Angpt-2, and VEGF plasmids alone or in combination (day 7), and 35 after surgical induction of ischemia (4 weeks after gene delivery). Immediately after surgery, perfusion was decreased in the ischemic vs. the non operated limb in all groups to a similar extent (I/NI: 0.25–0.32, Figure 5b). A modest recovery of ischemic hindlimb perfusion observed at day 7, prior to randomization to the various treatment groups. Rats receiving Angpt-2 transfer alone exhibited enhanced hindlimb perfusion by day 35, (Figure 5b; I/NI: 0.69±0.04 and 0.63±0.05, respectively, p<0.05 compared with control; 0.50±0.02). However, no significant improvement was seen within the group treated with the Angpt-1 plasmid (0.55±0.05), which was not different from the null-plasmid control group. Perfusion recovery in the ischemic limb was greater with Angpt-1 combined with VEGF (Figure 5c; 0.66±0.02) compared to Angpt-1 alone (p<0.05) but not different from VEGF alone (Figure 5c). In contrast, the combination Angpt-2 and VEGF gene transfer resulted in further improvement in ischemic limb perfusion (0.75±0.02, Figure 5c and d), significantly better than VEGF or Angpt-2 alone (p<0.05).

### Effect of Angiopoietins on collateral vessel formation

**Figure 6:**
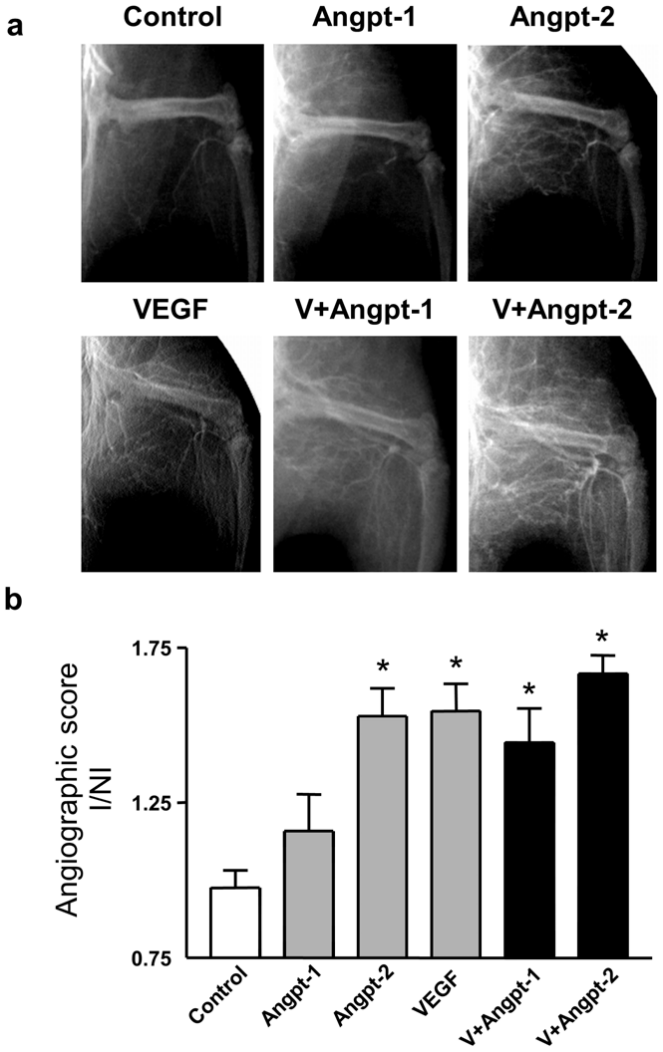
Hindlimb angiography and angiographic score quantitation. (a) Representative digital angiograms of bilateral limb vasculature in the arterial phase performed 35 days after hindlimb ischemia induction. (b) Angiographic score on post-operative day 35 was expressed as a ratio of collateral number in the ischemic limb to that in the non-ischemic limb. N = 5–10. * denotes p<0.01 versus control and Angpt-1.

Immediately after operative excision of femoral vasculature, no collaterals were detected in the thigh (adductor and vastus muscle groups) and there was only minimal distal leg filling. At day 35, some bridging collaterals originating from the internal iliac artery were visible in the thigh of null plasmid control animals with minimal distal filling, whereas collateral vessels and distal filling was much more pronounced in the animals treated with Angpt-2 and VEGF alone or in combination (Figure 6a). Collateralization was expressed as an angiographic score, defined as the ratio of vessel counts intersecting a grid in the ischemic versus non-ischemic hindlimb (Figure 6b). Treatment with VEGF or Angpt-2 alone or their combination resulted in a significant increase in collateral vessel formation compared with control animals, whereas no significant effect was seen with Angpt-1 alone. However, the combination of VEGF and Angpt-2 was not superior to VEGF or Angpt-2 alone.

### Enhanced perfusion recovery in transgenic mice overexpressing Angpt-2

**Figure 7:**
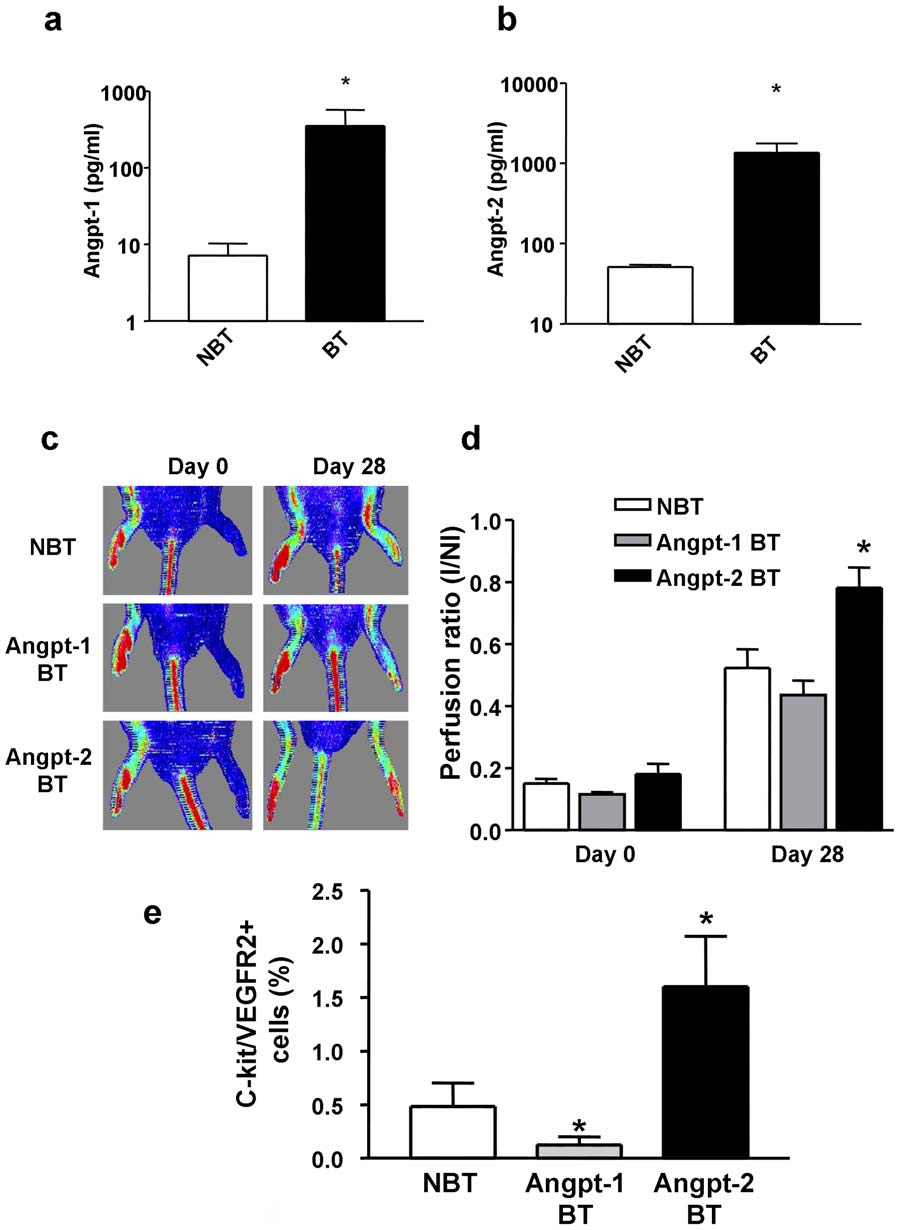
Confirmation of Angpt-1 and Angpt-2 overexpression in BT mice and its effects on ischemic neovascularization. ELISA quantitation of conditional hAngpt-1 (a) and hAngpt-2 (b) expression in binary transgenic (BT) and in control, non-binary transgenic (NBT) mice one week after withdrawal from doxycycline. (c) Representative laser doppler perfusion images performed serially immediately after femoral vasculature surgical excision (Day 0) and after four weeks are shown. (d) Quantitative serial evaluation of hindlimb perfusion in hAngpt-1 BT (n = 6), hAngpt-2 BT (n = 10) and NBT (n = 8) mice withdrawn from doxycycline on the same day as induction of hindlimb ischemia by femoral artery excision. * denotes p<0.01 versus Angpt-1 BT and NBT. (e) Mobilization of bone marrow-derived endothelial progenitor cells into the peripheral blood (VEGFR-2+/c-kit+) seven days after doxycycline withdrawal in NBT (n = 6), Angpt-1 BT (n = 4) and Angpt-2 BT (n = 5) mice. * denotes p<0.05 vs. NBT.

**Figure 8:**
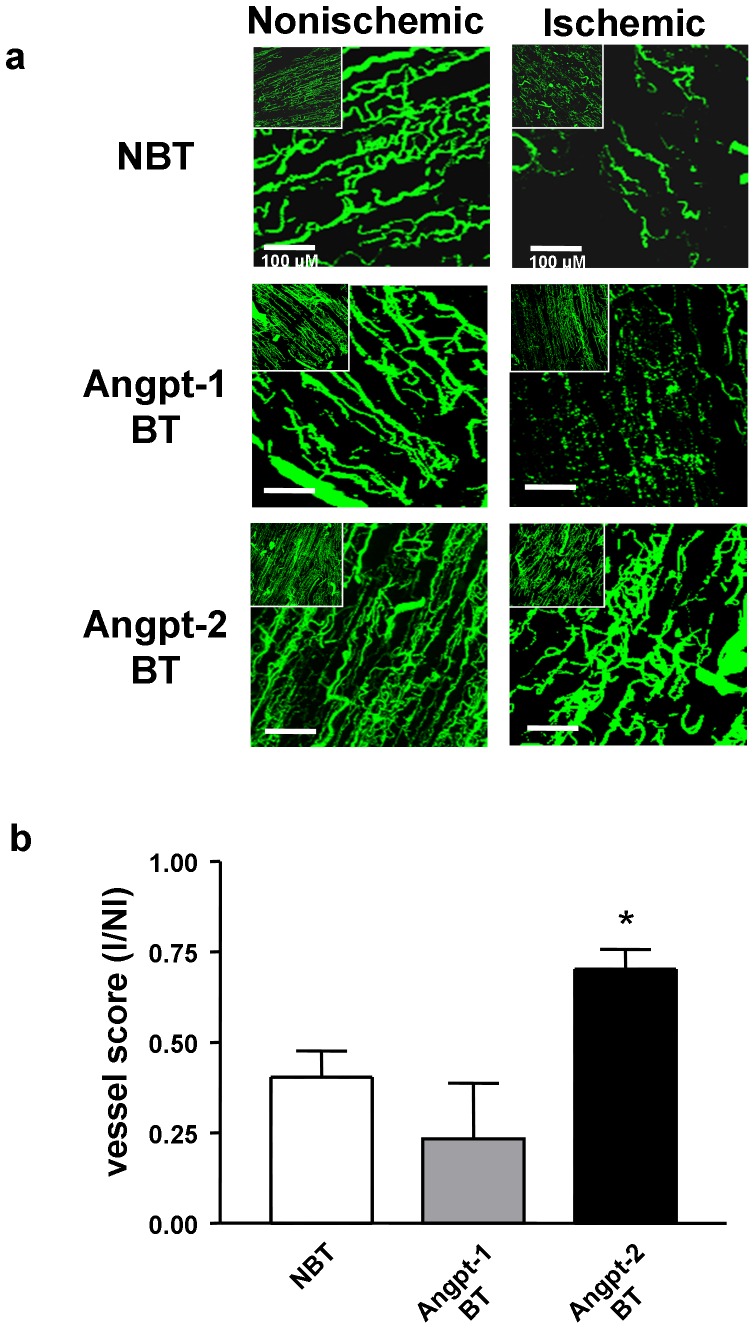
Fluorescence microangiography of hindlimb vasculature and neovessel quantitation. (a) Fluorescence microangiography derived representative stacked images of adductor muscle microvessels in ischemic and contralateral non-ischemic hindlimbs four weeks after surgical induction of hindlimb ischemia and withdrawal of doxycycline resulting in overexpression of Angpt-1 and Angpt-2. (b) Quantitation of adductor muscle neovascularization in response to hindlimb ischemia in binary transgenic mice overexpressing Angpt-1 (n = 3), Angpt-2 (n = 3) and non-BT littermates (n = 4). *denotes p<0.05 vs. Angpt-1 BT.

All binary transgenic (BT) animals were maintained on Dox in order to suppress transgene expression until the time of surgical induction of hindlimb ischemia. Measurement of human angiopoietin levels in plasma samples derived from binary transgenic (BT) mice confirmed conditional expression of hAngpt-1 and hAngpt-2 one week after Dox withdrawal (Figure 7a and b). Induction of transgene expression by withdrawal of Dox at the time of surgery significantly increased recovery of perfusion of the ischemic leg in hAngpt-2 BT mice at 4 weeks (I/NI: 0.78±0.07) compared with their NBT littermate controls (0.52±0.06, p<0.01), whereas there was no improvement seen in Angpt-1 BT mice (0.44±0.05, Figure 7c and d). Angpt-2 (but not Angpt-1) overexpression also increased the mobilization of c-kit/VEGFR2 double positive circulating progenitor cells in the peripheral blood (Figure 7e). Similarly, fluorescent microangiography (FMA) revealed improvement in functional vascularity of ischemic hindlimb muscle in the hAngpt-2 BT animals at 4 weeks post induction of ischemia (Figure 8a and b) compared to the hAngpt-1 BT and NBT mice.

## Discussion

To our knowledge, this is the first demonstration in the adult of the central role of the Tie2 receptor in the spontaneous neovascularization response to ischemia. We also show that the context-dependent partial Tie2 agonist/antagonist, Angpt-2, is a more potent proangiogenic and arteriogenic factor than Angpt-1 both in both rat and mouse hindlimb ischemia models, and may be a potentially useful therapeutic agent for the treatment of severe chronic ischemic syndromes.

Angpt-1 activates Tie2 to promote EC survival and quiescence and thus contributes to neovessel stabilitization and maturation during embryonic development, as well as the maintenance of postnatal vascular homeostasis [7]. Conversely, Angpt-2 is believed to function as an antagonist of Tie2 [8], releasing ECs from the tonic stabilizing effects of Angpt-1, and thus permitting efficient EC activation and plasticity in the response to classical aniogenic mediators, such as VEGF. However, recent reports suggest that Angpt-2 may acquire agonist activity under specific conditions, in particular during more prolonged exposure to Tie2 [13], [14]. Thus, this bimodal activity of Angpt-2 may be crucial in promoting efficient neovascularization by contributing to both the “activation” and “consolidation” phases of angiogenesis.

In order to better define the relevance of these changes for neovascularization, we used two complementary strategies to overexpresss Angpt-1 or Angpt-2 in the ischemic hindlimb: plasmid-based gene transfer and transgenic conditional overexpression. A consistent finding was the superior efficacy of Angpt-2 in restoring perfusion in the ischemic hindlimb compared with Angpt-1 using both approaches. Moreover, the effect of Angpt-2 on neovascularization was at least additive with VEGF in the rat model. The greater efficacy of Angpt-2 may be related to its bimodal functional activity, with an ability to both inhibit and activate Tie2 in a defined temporal sequence that closely corresponds to the time course of angiogenesis [14].

The lack of ability of Angpt-1 to induce neovascularization is consistent with a previous study showing that gene transfer of Angpt-1 alone or combined with VEGF using an adenoassociated virus vector failed to enhance neovascularization in the rat [23]. Furthermore, myocardial overexpression of Angpt-1 has been shown to inhibit VEGF-induced angiogenesis in a cardiac specific transgenic model [24]. However, several other studies have reported increased neovascularization in response to Angpt-1 [16], [25] but not Angpt-2. The reasons for the discrepancy between our study and the one by Shyu *et al.* may relate to species utilized and the “dose” of plasmid DNA used, which was very different in the two studies [16]. Shyu *et al.* injected 125 µg of plasmid-encoding DNA into the larger volume of the rabbit hindlimb compared to our study utilizing 500 µg in the smaller rat hindlimb. Finally, it is likely that there were differences in the degree of induced ischemia between the two models given the greater size of rabbit hindlimb and species differences in pre-existing collaterals.

There have been relatively few studies that have examined the ability of Angpt-2 either alone or in combination with VEGF to induce angiogenesis, and these have produced conflicting results. A previous report showed that increased expression of VEGF and Angpt-2 in transgenic mice had a synergistic effect on enhancing capillary number in the myocardium [24], though this was in the absence of a physiologically relevant ischemic stimulus. In contrast, a report by Reiss *et al.* [17] using a binary transgenic model system similar to ours showed that targeted vascular overexpression of Angpt-2 reduced perfusion recovery in the ischemic hindlimb, a finding seemingly opposite of that described in the present report. However, different targeting strategies were used in these two studies, which likely resulted in marked differences in local concentrations of Angpt-2 in the ischemic limb. In the present study, the transgene was targeted to the liver using a hepatic-specific promoter, and delivery of Angpt-2 to the ischemic hindlimb was entirely dependent on hepatic release and transport via the circulation, thus mimicking the effects of systemic administration of a therapeutic protein. In contrast, the earlier report targeted the Angpt-2 transgene directly to the vascular endothelium-specific Tie1 promoter, thus Angpt-2 was directly overexpressed locally within the vascular endothelium of the hindlimb. Although both studies reported similar increases in circulating Angpt-2 levels, endothelial-targeted expression would result in far greater local vascular levels, and these very high concentrations of Angpt-2 have been shown to have potentially deleterious effects on vascular integrity and function [26], [27]. In contrast, in our transgenic system, the site of Angpt-2 generation and its action were segregated, and thus any effects on the ischemic limb are solely attributable to the effects of increased circulating protein. Similarly, the gene transfer model utilized direct plasmid DNA injection that results in a relatively modest degree of muscle transfection [28], [29], and which does not result detectable increases in circulating levels [30].

In support of the possibility that differences in local vascular Angpt-2 levels could be responsible for the discrepant findings between our study and that of Reiss et al, this group showed a clear “dose-dependent” effect such that mice exhibiting highest expression levels showed the greatest impairment in angiogenesis. Based on our results, we would suggest that this dose-effect relationship in fact reverses at more physiological concentrations, such that these lower local vascular Angpt-2 levels promote angiogensis perfusion recovery. Indeed, this was confirmed by a recent report showing reduced blood flow recovery in the ischemic hindlimb model after specific inhibition of endogenous Angpt-2-Tie2 interactions, using a selective Angpt-2 antagonist [20]. This is also supported by a recent report showing the superiority of Angpt-2 compared with Angpt-1 inhibition in inhibiting tumor angiogenesis [31]. Moreover, Angpt-2 has recently been shown to reduce atherogenesis by an NO/eNOS-dependent mechanism [32], which is also an important pathway in angiogenesis [33], [34]. In this context, the reduction in basal eNOS expression in Tie2-deficient mice might be causally linked to the reduction in neovascularization capacity demonstrated in this model.

The role of Angpt-1 and Tie2 in the mobilization of bone marrow-derived stem and progenitor cells is also controversial. Increased levels of circulating progenitor cells have been reported after administration of Angpt-1 suggesting that, like VEGF, Angpt-1 can induce bone marrow mobilization [35]. However, this finding is not consistent with a more recent report showing that Angpt-1 promotes tight interactions between hematopoietic stem cells and stromal cells, and thus contributes to the sequestration of stem and progenitor cells within the bone marrow niche [36]. Our findings with angiopoietin overexpressing transgenic mice are consistent with the latter, since induction of Angpt-1 transgene expression reduced circulating levels of VEGFR-2+/c-kit+ cells, whereas Angpt-2 overexpression increased circulating levels of progenitor cells. To our knowledge, a role for Angpt-2 as a potential mediator of progenitor cell mobilization from the bone marrow has not been previously reported. Thus, these novel findings suggest that the mechanism by which Angpt-2 improves blood flow recovery in the ischemic hindlimb model may involve both local effects on the peripheral vasculature as well as central actions on the mobilization of bone marrow stem and progenitor cells, which may act in concert to enhance neovascularization and perfusion of ischemic tissue.

In conclusion, these data demonstrate that Angpt-2, but not Angpt-1, is a potent mediator of angiogenesis and collateralization in the ischemic hindlimb model when expressed at levels that are physiologically relevant. The superiority of Angpt-2 may be due to its bifunctional role as a modulator of Tie2 activation, incorporating both antagonist and agonist activities and thus contributing to the initial increase in vessel plasticity and sprouting, while enhancing maturation in the later phases of neovascularization [37]. These data support further exploration of the use of Angpt-2 as a potential proangiogenesis agent.

## Materials and Methods

### Animals: Rodent Hindlimb Ischemia Model

All animal protocols were approved by the Institutional Animal Care and Use Committee at St. Michael's Hospital. Male Sprague-Dawley rats (500–650 gm) were anesthetized with intraperitoneal Ketamine (200 mg/kg) and Xylazine (100 mg/kg). Surgery was performed to induce a model of chronic unilateral hindlimb ischemia. After a lower abdominal midline incision, the entire femoral artery was exposed and excised after ligation of the inferior epigastric, deep femoral, lateral circumflex and superficial epigastric arteries. Details for the preparation of the mouse hindlimb ischemia model are provided in detail in Materials and Methods S1.

### Gene Transfer Studies

Rats underwent surgery (day 0) to induce hindlimb ischemia. On day 7, animals were divided into six groups that received intramuscular injections of plasmid DNA encoding human *Angpt-1*(n = 7), *Angpt-2* (n = 8), *VEGF* (n = 9) or combination of *Angpt-1*+*VEGF* (n = 8) and *Angpt-2*+*VEGF* (n = 7). Control animals received null (empty) plasmid (n = 9). Serial, non-invasive assessment of perfusion by laser doppler perfusion imaging was performed on day 0, 7, 14 and 35. Animals were sacrificed at day 35. At sacrifice, angiography was done to assess for degree of collateralization. In a separate groups of rats, the gene expression profile of *Angpt-1*, *Angpt-2*, *VEGF* following 1 (n = 5) and 28 (n = 5) days of hindlimb ischemia was assessed from muscles taken from ischemic and contralateral non-ischemic limbs for real-time reverse transcriptase polymerase chain reaction (RT-PCR) analysis.

### Intramuscular Gene Transfer

Human *Angpt-1*, *Angpt-2* and *VEGF*_164_ cDNA were subcloned into cytomegalovirus (CMV) promoter-driven and ampicillin-resistance gene containing, mammalian cell expression vectors pFLAG, pCRUni3.1 and pVax respectively. On day 7, plasmid DNA was delivered by five injections in the ischemic leg, namely, two into the adductor, one into vastus medialis and two sites in the gastrocnemius muscle using a 1-mL syringe and a 30-gauge needle. A total of 500 µg (single gene transfer) or 1000 µg (combination gene transfer) of plasmid DNA in a constant volume of 1000 µL of saline was injected. In mouse studies, human eNOS was subcloned into the plasmid pVax. On day 7 plasmid DNA (150 µg in 100 µL saline) was delivered via injection (BD Ultrafine Insulin syringe) similar to gene transfer studies in the rats.

### Laser Doppler Pefusion Imaging

Both rats and mice were anesthetized with Ketamine (200 mg/kg) and Xylazine (10 mg/kg). Hair was removed using an electric shaver. Serial, non-invasive assessment of ischemic limb microvascular perfusion was performed in triplicate and in a blinded manner using the LDPI system (Moor instruments, United Kingdom) after placing all mice on a homeothermic heating pad maintained at 37°C. Identical regions equal in area encompassing the distal leg (entire foot) of both ischemic and contralateral non-ischemic limbs were assessed for quantification of perfusion using the LDPI image processing software (v5.0). To minimize data variation possibly secondary to ambient temperature changes all perfusion data were expressed as a ratio of operated ischemic (I) to non-operated, non-ischemic (NI) limb perfusion.

### Angiographic assessment of collateral circulation

Rats were anesthetized with Ketamine (200 mg/kg) and Xylazine (10 mg/kg). An angiocatheter (24G, Becton-Dickinson, Franklin Lakes, NJ) was inserted into the abdominal aorta, below the renal arteries and above the iliac artery bifurcations as described in detail in Materials and Methods S1.

### Fluorescent microangiography of hindlimb vasculature

A catheter was inserted into the mouse abdominal aorta and then flushed with heparinized heparinized saline followed by right atrium venting. A warmed (45°C) solution of 1% low melting point agarose (Sigma) containing 0.2 µm yellow-green fluorescent microspheres (505/515 nm excitation-emission, Life Technologies, Carlsbad, CA) was then slowly infused into the aorta at a constant rate resulting in bilateral hindlimb perfusion. The animal was immediately placed on ice and cooled to promote solidification of the agarose solution. Harvested adductor muscle was then placed in OCT on dried ice and subsequently sectioned (100 µm) using an oscillating blade microtome (Leica, Richmond Hill, Canada). Confocal microscopy (Bio-Rad Laboratories, Mississauga, Canada) was used to produce a Z stack of images that were projected in order to quantify vessel number using automated software (IPTK analysis software, Reindeer Graphics Inc., Asheville, NC). This technique has previously been used to quantify pulmonary vasculature density in a rat experimental model of pulmonary hypertension [38].

### Tie2 Deficient Mice

The generation of Tie2 knockout mice by targeted deletion of the *Tie2* gene has been previously reported [39]. Wheras the homozygous null mutation is embryonic lethal, heterozygous Tie2-defiicent mice are viable with no overt phenotype. Genotypes of wild-type (WT, Tie2+/+) and heterozygote deficient (Tie2^+/−^) mice were determined by PCR. Mice were on a CD1 genetic strain background. All animal protocols were approved by the Institutional Animal Care and Use Committee at St. Michael's Hospital. Heterozygote deficient and wild-type littermates were used at 12–18 weeks of age. Genomic DNA was extracted from mouse ear-notch samples and PCR was performed using the buffers and PCR reaction mix from the REDExtract-N-Amp™ Tissue PCR Kit, following manufacturer's instructions (Sigma-Aldrich, St. Louis, MO). Briefly, samples were incubated in a 1∶4 mixture of the tissue preparation solution and extraction solution for 10 minutes at room temperature, heated to 95°C for 3 minutes, and then mixed with a neutralization solution to deactivate inhibitory substances prior to PCR. A 0.5 µm mixture of the following primers was used in the PCR to detect the transgene-inserted Tie2 (∼350 bp) and endogenous Tie2 (∼150 bp): Forward (endogenous): 5′ - AGC GCG TGG ACC ATG CGA GC - 3′, Reverse (endogenous): 5′ - CCA TTG CTC AGC GGT GCT GTC CAT - 3′, and Forward (transgene): 5′ - AGG AGC AAG CTG ACT CCA CAG - 3′. The PCR thermal cycling parameters included an initial denaturation at 94°C for 3 min., followed by 30 cycles consisting of denaturation at 95°C for 30 sec., annealing at 54°C for 40 sec., and extension at 72°C for 30 sec. Following this, a final extension at 72°C for 10 min. was performed and the amplified DNA resolved in a 1.5% agarose gel.

### Conditional Angiopoietin Transgenic Mice

**Figure 9:**
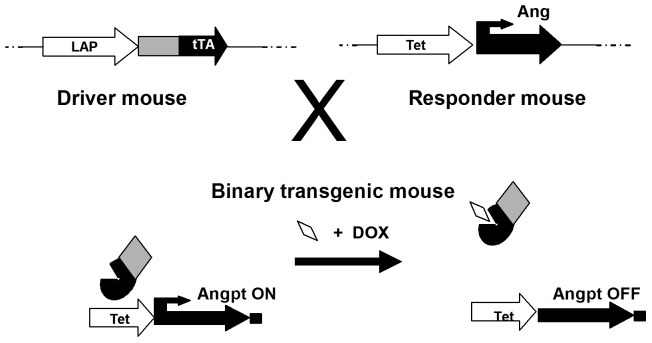
Three independent lines of transgenic mice were used to induce conditional overexpression of human Angpt-1 (hAngpt-1) and human Angpt-2 (hAngpt-2). The tetracycline-responsive transactivator (tTA), expressed from the LAP promoter (driver line) associates with the tTA binding site (Tet^OS^) located upstream of the hAngpt-1 or hAngpt-2 cDNA (responder line). In the presence of the tetracycline analog, doxycycline (DOX), hAngpt-1 or hAngpt-2 expression is suppressed. In the absence of DOX, hAngpt-1 or hAngpt-2 are expressed.

Two binary transgenic (BT) mouse models in a CD1 background were generated with conditional overexpression of human Angpt-1 (hAngpt-1) or human Angpt-2 (hAngpt-2), respectively, driven by a tetracycline (doxycycline) responsive, liver specific, Lap-1 promoter (Figure 9) [40], as is more fully described in the expanded in Materials and Methods S1. BT (Lap∶hAngpt-1 and Lap∶hAngpt-2) and single transgenic (ST: Lap, hAngpt-1 or hAngpt-2) mice were maintained on drinking water containing Dox (100 µg/mL) in a 5% drinking solution from conception up to adulthood. Mice (16–24 weeks old) were withdrawn from Dox at the time of surgical induction of unilateral hindlimb ischemia (day 0), as previously described. Microvascular hindlimb perfusion was assessed by laser doppler perfusion imaging on day 0 as well as 35 and expressed as a ratio of ischemic to non-ischemic limb perfusion. Mice were sacrificed on day 35.

### Measurement of human Angpt-1 and Angpt-2

The concentration of hAngpt-1 and hAngpt-2 in the serum of transgenic mice was measured with enzyme-linked immunosorbent assay (ELISA) human Angpt-1 and Angpt-2 kits (R&D Systems, Minneapolis, MN) as per the manufacturer's instructions. Blood collected by cardiac puncture from BT∶hAngpt-1 and BT∶hAngpt-2 overexpressing mice was placed into ethylenediaminetetraacetic acid (EDTA)-lined tubes and spun at 1000 g for 15 minutes at 2–5°C within 30 minutes of collection. Plasma from BT∶hAngpt-1 expressing mice was spun again at 10,000 g for 10 minutes at 2–5°C to ensure complete platelet removal prior to assay and stored at −70°C.

### RNA Extraction and Quantitative Reverse Transcription – Polymerase Chain Reaction (qRT-PCR)

Total RNA was extracted from mouse liver as well as rat ischemic hindlimb and contralateral limb adductor and gastrocnemius muscles using Trizol extraction (Life Technologies, Carlsbad, CA) as per manufacturers' protocols. Extracted RNA (1 µg) and Omniscript Reverse Transcriptase (Qiagen, Hilden, Germany) were used in RT reactions that were diluted 1/20. Each diluted RT was then used in PCRs in duplicates to assess the RNA abundance of rat Angpt-1, Angpt-2, VEGF and Tie2 using the SYBR Green PCR Master Mix and the ABI PRISM 7900HT sequence detection system (Applied Biosystems, Life Technologies, Carlsbad, CA). Further detail including a complete list of the sequences for primers utilized in these studies is located in Materials and Methods S1.

### Peripheral Blood Cell Mobilization

The profile of circulating progenitor cells was analyzed in transgenic mice 7 days after doxycycline withdrawal. Briefly, animals were anesthetized and underwent cardiac puncture for blood withdrawal (800–1000 µL) collected in K_2_EDTA tubes (BD Biosciences, Mississauga, Canada). Following red blood cell lysis with Lysis buffer (Sigma), flow cytometry analysis for c-kit (CD117) and VEGFR-2 were performed using a FC500 flow cytometer (Beckman Coulter, Brea, CA).

### Statistical Analysis

Data was expressed as mean ± SEM. Comparison between two means was done using the Student's unpaired t-test. For all other comparisons, one-way ANOVA with a post-hoc Neuman-Keuls test for multiple comparisons was used. Significance was set at p<0.05.

## Supporting Information

Materials and Methods S1 Expanded list of materials and methods used in this study.
(DOC)

## References

[1] Dumont DJ, Yamaguchi TP, Conlon RA, Rossant J, Breitman ML (1992) tek, a novel tyrosine kinase gene located on mouse chromosome 4, is expressed in endothelial cells and their presumptive precursors. Oncogene 7: 1471–1480. 1630810

[2] Sato TN, Tozawa Y, Deutsch U, Wolburg-Buchholz K, Fujiwara Y, et al (1995) Distinct roles of the receptor tyrosine kinases Tie-1 and Tie-2 in blood vessel formation. Nature 376: 70–74. 7596437 10.1038/376070a0

[3] Suri C, Jones PF, Patan S, Bartunkova S, Maisonpierre PC, et al (1996) Requisite role of angiopoietin-1, a ligand for the TIE2 receptor, during embryonic angiogenesis. Cell 87: 1171–1180. 8980224 10.1016/s0092-8674(00)81813-9

[4] Carmeliet P, Ferreira V, Breier G, Pollefeyt S, Kieckens L, et al (1996) Abnormal blood vessel development and lethality in embryos lacking a single VEGF allele. Nature 380: 435–439. 8602241 10.1038/380435a0

[5] Thurston G, Suri C, Smith K, McClain J, Sato TN, et al (1999) Leakage-resistant blood vessels in mice transgenically overexpressing angiopoietin-1. Science 286: 2511–2514. 10617467 10.1126/science.286.5449.2511

[6] Thurston G, Rudge JS, Ioffe E, Zhou H, Ross L, et al (2000) Angiopoietin-1 protects the adult vasculature against plasma leakage. Nat Med 6: 460–463. 10742156 10.1038/74725

[7] Yancopoulos GD, Davis S, Gale NW, Rudge JS, Wiegand SJ, et al (2000) Vascular-specific growth factors and blood vessel formation. Nature 407: 242–248. 11001067 10.1038/35025215

[8] Maisonpierre PC, Suri C, Jones PF, Bartunkova S, Wiegand SJ, et al (1997) Angiopoietin-2, a natural antagonist for Tie2 that disrupts in vivo angiogenesis. Science 277: 55–60. 9204896 10.1126/science.277.5322.55

[9] Tanaka S, Mori M, Sakamoto Y, Makuuchi M, Sugimachi K, et al (1999) Biologic significance of angiopoietin-2 expression in human hepatocellular carcinoma. J Clin Invest 103: 341–345. 9927494 10.1172/JCI4891 PMC407900

[10] Peters KG (1998) Vascular endothelial growth factor and the angiopoietins: working together to build a better blood vessel. Circ Res 83: 342–343. 9710128 10.1161/01.res.83.3.342

[11] Lobov IB, Brooks PC, Lang RA (2002) Angiopoietin-2 displays VEGF-dependent modulation of capillary structure and endothelial cell survival in vivo. Proc Natl Acad Sci U S A 99: 11205–11210. 12163646 10.1073/pnas.172161899 PMC123234

[12] Goede V, Schmidt T, Kimmina S, Kozian D, Augustin HG (1998) Analysis of blood vessel maturation processes during cyclic ovarian angiogenesis. Lab Invest 78: 1385–1394. 9840613

[13] Kim I, Kim JH, Moon SO, Kwak HJ, Kim NG, et al (2000) Angiopoietin-2 at high concentration can enhance endothelial cell survival through the phosphatidylinositol 3′-kinase/Akt signal transduction pathway. Oncogene 19: 4549–4552. 11002428 10.1038/sj.onc.1203800

[14] Teichert-Kuliszewska K, Maisonpierre PC, Jones N, Campbell AI, Master Z, et al (2001) Biological action of angiopoietin-2 in a fibrin matrix model of angiogenesis is associated with activation of Tie2. Cardiovasc Res 49: 659–670. 11166279 10.1016/s0008-6363(00)00231-5

[15] Asahara T, Chen D, Takahashi T, Fujikawa K, Kearney M, et al (1998) Tie2 receptor ligands, angiopoietin-1 and angiopoietin-2, modulate VEGF-induced postnatal neovascularization. Circ Res 83: 233–240. 9710115 10.1161/01.res.83.3.233

[16] Shyu KG, Manor O, Magner M, Yancopoulos GD, Isner JM (1998) Direct intramuscular injection of plasmid DNA encoding angiopoietin-1 but not angiopoietin-2 augments revascularization in the rabbit ischemic hindlimb. Circulation 98: 2081–2087. 9808608 10.1161/01.cir.98.19.2081

[17] Reiss Y, Droste J, Heil M, Tribulova S, Schmidt MH, et al (2007) Angiopoietin-2 impairs revascularization after limb ischemia. Circ Res 101: 88–96. 17540977 10.1161/CIRCRESAHA.106.143594

[18] Hackett SF, Ozaki H, Strauss RW, Wahlin K, Suri C, et al (2000) Angiopoietin 2 expression in the retina: upregulation during physiologic and pathologic neovascularization. J Cell Physiol 184: 275–284. 10911358 10.1002/1097-4652(200009)184:3<275::AID-JCP1>3.0.CO;2-7

[19] Gale NW, Thurston G, Hackett SF, Renard R, Wang Q, et al (2002) Angiopoietin-2 is required for postnatal angiogenesis and lymphatic patterning, and only the latter role is rescued by Angiopoietin-1. Dev Cell 3: 411–423. 12361603 10.1016/s1534-5807(02)00217-4

[20] Tressel SL, Kim H, Ni CW, Chang K, Velasquez-Castano JC, et al (2008) Angiopoietin-2 stimulates blood flow recovery after femoral artery occlusion by inducing inflammation and arteriogenesis. Arterioscler Thromb Vasc Biol 28: 1989–1995. 18772493 10.1161/ATVBAHA.108.175463 PMC2613177

[21] Stabile E, Burnett MS, Watkins C, Kinnaird T, Bachis A, et al (2003) Impaired arteriogenic response to acute hindlimb ischemia in CD4-knockout mice. Circulation 108: 205–210. 12821542 10.1161/01.CIR.0000079225.50817.71

[22] Rutherford RB, Baker JD, Ernst C, Johnston KW, Porter JM, et al (1997) Recommended standards for reports dealing with lower extremity ischemia: revised version. J Vasc Surg 26: 517–538. 9308598 10.1016/s0741-5214(97)70045-4

[23] Arsic N, Zentilin L, Zacchigna S, Santoro D, Stanta G, et al (2003) Induction of functional neovascularization by combined VEGF and angiopoietin-1 gene transfer using AAV vectors. Mol Ther 7: 450–459. 12727107 10.1016/s1525-0016(03)00034-0

[24] Visconti RP, Richardson CD, Sato TN (2002) Orchestration of angiogenesis and arteriovenous contribution by angiopoietins and vascular endothelial growth factor (VEGF). Proc Natl Acad Sci U S A 99: 8219–8224. 12048246 10.1073/pnas.122109599 PMC123048

[25] Yamauchi A, Ito Y, Morikawa M, Kobune M, Huang J, et al (2003) Pre-administration of angiopoietin-1 followed by VEGF induces functional and mature vascular formation in a rabbit ischemic model. J Gene Med 5: 994–1004. 14601137 10.1002/jgm.439

[26] Fiedler U, Augustin HG (2006) Angiopoietins: a link between angiogenesis and inflammation. Trends Immunol 27: 552–558. 17045842 10.1016/j.it.2006.10.004

[27] Fiedler U, Reiss Y, Scharpfenecker M, Grunow V, Koidl S, et al (2006) Angiopoietin-2 sensitizes endothelial cells to TNF-alpha and has a crucial role in the induction of inflammation. Nat Med 12: 235–239. 16462802 10.1038/nm1351

[28] Wolff JA, Malone RW, Williams P, Chong W, Acsadi G, et al (1990) Direct gene transfer into mouse muscle in vivo. Science 247: 1465–1468. 1690918 10.1126/science.1690918

[29] Tripathy SK, Svensson EC, Black HB, Goldwasser E, Margalith M, et al (1996) Long-term expression of erythropoietin in the systemic circulation of mice after intramuscular injection of a plasmid DNA vector. Proc Natl Acad Sci U S A 93: 10876–10880. 8855275 10.1073/pnas.93.20.10876 PMC38250

[30] Comerota AJ, Throm RC, Miller KA, Henry T, Chronos N, et al (2002) Naked plasmid DNA encoding fibroblast growth factor type 1 for the treatment of end-stage unreconstructible lower extremity ischemia: preliminary results of a phase I trial. J Vasc Surg 35: 930–936. 12021709 10.1067/mva.2002.123677

[31] Coxon A, Bready J, Min H, Kaufman S, Leal J, et al (2010) Context-dependent role of angiopoietin-1 inhibition in the suppression of angiogenesis and tumor growth: implications for AMG 386, an angiopoietin-1/2-neutralizing peptibody. Mol Cancer Ther 9: 2641–2651. 20937592 10.1158/1535-7163.MCT-10-0213 PMC4430860

[32] Ahmed A, Fujisawa T, Niu XL, Ahmad S, Al-Ani B, et al (2009) Angiopoietin-2 confers Atheroprotection in apoE−/− mice by inhibiting LDL oxidation via nitric oxide. Circ Res 104: 1333–1336. 19461044 10.1161/CIRCRESAHA.109.196154 PMC2938017

[33] Murohara T, Asahara T, Silver M, Bauters C, Masuda H, et al (1998) Nitric oxide synthase modulates angiogenesis in response to tissue ischemia. J Clin Invest 101: 2567–2578. 9616228 10.1172/JCI1560 PMC508846

[34] Papapetropoulos A, Garcia-Cardena G, Madri JA, Sessa WC (1997) Nitric oxide production contributes to the angiogenic properties of vascular endothelial growth factor in human endothelial cells. J Clin Invest 100: 3131–3139. 9399960 10.1172/JCI119868 PMC508526

[35] Rabbany SY, Heissig B, Hattori K, Rafii S (2003) Molecular pathways regulating mobilization of marrow-derived stem cells for tissue revascularization. Trends Mol Med 9: 109–117. 12657432 10.1016/s1471-4914(03)00021-2

[36] Bazzoni G, Dejana E (2004) Endothelial cell-to-cell junctions: molecular organization and role in vascular homeostasis. Physiol Rev 84: 869–901. 15269339 10.1152/physrev.00035.2003

[37] Schaper W, Scholz D (2003) Factors regulating arteriogenesis. Arterioscler Thromb Vasc Biol 23: 1143–1151. 12676799 10.1161/01.ATV.0000069625.11230.96

[38] Zhao YD, Courtman DW, Deng Y, Kugathasan L, Zhang Q, et al (2005) Rescue of monocrotaline-induced pulmonary arterial hypertension using bone marrow-derived endothelial-like progenitor cells: efficacy of combined cell and eNOS gene therapy in established disease. Circ Res 96: 442–450. 15692087 10.1161/01.RES.0000157672.70560.7b

[39] Dumont DJ, Gradwohl G, Fong GH, Puri MC, Gertsenstein M, et al (1994) Dominant-negative and targeted null mutations in the endothelial receptor tyrosine kinase, tek, reveal a critical role in vasculogenesis of the embryo. Genes Dev 8: 1897–1909. 7958865 10.1101/gad.8.16.1897

[40] Haninec AL, Voskas D, Needles A, Brown AS, Foster FS, et al (2006) Transgenic expression of Angiopoietin 1 in the liver leads to changes in lymphatic and blood vessel architecture. Biochem Biophys Res Commun 345: 1299–1307. 16730659 10.1016/j.bbrc.2006.04.149

