# Supplementary material for: Tie2-Dependent Neovascularization of the Ischemic Hindlimb Is Mediated by Angiopoietin-2
Source: PLoS One. 2012 Sep 25;7(9):e43568. doi: 10.1371/journal.pone.0043568 (PMC3458045; doi:10.1371/journal.pone.0043568)
Supplement: Materials and Methods S1 — Expanded list of materials and methods used in this study. (DOC) [file pone.0043568.s001.doc]

**Online Supplement**

**Supplementary Materials and Methods**

***Hindlimb Ischemia Model:*** Two models of surgically-induced unilateral hindlimb ischemia in the mouse were utilized: a critical limb ischemia model, characterized by limb necrosis and autoamputation and a model of moderate chronic limb ischemia that is without tissue infarction and necrosis [1, 2]. In the critical limb ischemia model, mice were anesthetized with intraperitoneal Ketamine (200mg/kg) and Xylazine (10mg/kg). After a lower abdominal midline incision, the entire femoral artery was exposed and isolated from the level of the inguinal ligament to its bifurcation into saphenous and popliteal arteries and excised. Limb autoamputation was defined as loss of the distal forefoot due to profound ischemia characterized by tissue necrosis. Chronic hindlimb ischemia was generated by ligation and partial excision of the common femoral artery resulting in a consistently reproducible model of moderate chronic ischemia, independent of tissue loss, that allows for the serial evaluation of perfusion restoration in response to ischemia. The wound was closed with 3-0 Vicryl. Consequently the blood flow and perfusion to the ischemic lower limb becomes dependent on collateral flow derived from the internal iliac artery. The rodent chronic ischemic hindlimb is a consistently reproducible model of moderate chronic ischemia without infarction or tissue loss that allows for the study of ischemic neovascularization. The induction of hindlimb ischemia increases hypoxia inducible factor-1α (HIF-1α) expression in the ischemic hindlimb (data not shown).

***In vivo assessment of limb function:*** Semiquantitative assessment of impaired use of the ischemic limb was perforned serially using the following classification: 3=dragging of foot, 2=no dragging but no plantar flexion, 1=plantar flexion, and 0=flexing the toes to resist gentle traction on the tail [3, 4].

***Angiographic assessment of collateral circulation:***

Animals were euthazined with sodium pentobarbital overdose and hindlimb skin removed to avoid superficial vessel image artifacts. The lower hindlimb was perfused with heparinized saline (10 U/ml). Post-mortem angiography was performed utilizing Omnipaque (Amersham) as angiographic contrast hand infused at a rate of 0.5 ml/s for 20 seconds. The recorded film of the pelvis and both hindlimbs was analyzed using Image J software (NIH) to calculate an angiographic score of an area encompassing the thigh from the hip joint to the knee. Specifically, a grid was laid over an image of the arterial filling phase vasculature and the number of collateral vessel intersections with the grid over a defined, bilaterally equal, area was counted [5]. Angiographic score was expressed as a ratio of the operated ischemic leg to non-operated, non-ischemic leg counts. Therefore, each animal served as its’ own control.

***Transgenic mice:*** “Driver” mice harboured the tetracycline-responsive transactivator (tTA) under the control of the Lap promoter and “responder” mice were engineered with the human (h) Angpt-1 or Angpt-2 cDNA downstream of a tetracycline response promoter (Tet^OS^). In the absence of the tetracycline analog, doxycycline (DOX, Sigma), hAngpt-1 or hAngpt-2 is expressed and in the presence of Dox hAngpt-1 or hAngpt-2 expression is suppressed (i.e. Dox-off) (Figure 9). The mouse driver (Lap) and responder (hAngpt-1 and hAngpt-2) lines were generated as described previously [6, 7]. Single transgenic (ST) Lap mice were individually crossed with hAngpt-1 or hAngpt-2 ST mice and offspring were genotyped by PCR using DNA extracted from tail biopsies. DNA was prepared and PCR performed using primers previously described [8]. Non-BT littermates served as experimental controls. Mice were on a CD1 genetic strain background.

***RNA Extraction and Quantitative Reverse Transcription – Polymerase Chain Reaction (qRT-PCR):*** Endogenous relative rat Angpt-1, Angpt-2, Tie2 and VEGF RNA expression was calculated in comparison to rat HPRT RNA expression using Pfaffl’s formula [9]. Gene expression was expressed as a ratio of ischemic limb to non-ischemic as outlined in the formula:

| Ratio of relative expression = | (E_gene of interest_)[CPgene of interest (non-ischemic limb) – CPgene of interest (ischemic limb)] |
| --- | --- |
|  | (E_reference gene_)[CPreference gene(non-ischemic limb) – CPreference gene (ischemic limb)] |

Primer efficiencies (E) were determined by dilution curve analysis and CP is defined as the crossing point or threshold cycle.

***qRT-PCR primers:*** rat Angpt1 (sense primer: 40405’‑AGATACAACAGAATGCGGTTCAAA‑3’, antisense primer: 5’‑TGAGACAAGAGGCTGGTTCCTAT‑3’), rat Angpt2 (sense primer: 5’‑TGGCTGGGCAACGAGTTT‑3’, antisense primer: 5’‑TGGATCTTCAGCACGTAGCG‑3’), rat VEGF (sense primer: 5’‑TTCAAGCCGTCCTGTGTGC‑3’, antisense primer: 5’‑TCCAGGGCTTCATCATTGC‑3’), rat MCP-1(sense primer: 5’-CAGATGCAGTTAATGCCCCAC’, antisense primer: 5’-AGCCGACTCATGGGATCAT-3’), rat 18S (sense primer: 5’-GACGATCAGATACCGTCGTAGTTC-3’, antisense primer: 5’-GTTTCAGCTTTGCAACCATACTCC-3’), rat Hypoxanthine-Guanine Phosphoribosyl Transferase (HPPRT) (sense primer: 5’-GTCAACGGGGGACATAAAAG-3’, antisense primer: 5’-TGCATTGTTTTACCAGTGTCAA-3’), mouse MCP-1 (sense primer: 5’-GGCTGGAGAGCTACAAGAGG-3’, antisense primer: 5’-TCTTGAGCTTGGTGACAAAAAC-3’) and mouse β-actin (sense primer: 5’-AAGGCCAACCGTGAAAAGAT-3’, antisense primer: 5’-GTGGTACGACCAGAGGCATAC-3’)

**Supplementary** **References**

1. Masaki, I*, et al.* (2002). Angiogenic gene therapy for experimental critical limb ischemia: acceleration of limb loss by overexpression of vascular endothelial growth factor 165 but not of fibroblast growth factor-2. *Circ Res* **90**: 966-973.

2. Couffinhal, T, Silver, M, Zheng, LP, Kearney, M, Witzenbichler, B, and Isner, JM (1998). Mouse model of angiogenesis. *Am J Pathol* **152**: 1667-1679.

3. Stabile, E*, et al.* (2003). Impaired arteriogenic response to acute hindlimb ischemia in CD4-knockout mice. *Circulation* **108**: 205-210.

4. Rutherford, RB*, et al.* (1997). Recommended standards for reports dealing with lower extremity ischemia: revised version. *J Vasc Surg* **26**: 517-538.

5. Yamauchi, A*, et al.* (2003). Pre-administration of angiopoietin-1 followed by VEGF induces functional and mature vascular formation in a rabbit ischemic model. *J Gene Med* **5**: 994-1004.

6. Kistner, A*, et al.* (1996). Doxycycline-mediated quantitative and tissue-specific control of gene expression in transgenic mice. *Proc Natl Acad Sci U S A* **93**: 10933-10938.

7. Furth, PA*, et al.* (1994). Temporal control of gene expression in transgenic mice by a tetracycline-responsive promoter. *Proc Natl Acad Sci U S A* **91**: 9302-9306.

8. Sarao, R, and Dumont, DJ (1998). Conditional transgene expression in endothelial cells. *Transgenic Res* **7**: 421-427.

9. Pfaffl, MW (2001). A new mathematical model for relative quantification in real-time RT-PCR. *Nucleic Acids Res* **29**: e45.

10. Scholz, D*, et al.* (2002). Contribution of arteriogenesis and angiogenesis to postocclusive hindlimb perfusion in mice. *J Mol Cell Cardiol* **34**: 775-787.
